# Fabrication and Properties of Carbon Fibers

**DOI:** 10.3390/ma2042369

**Published:** 2009-12-16

**Authors:** Xiaosong Huang

**Affiliations:** Chemical Sciences & Materials Systems Laboratory, General Motors Research & Development Center, Mail Code 480-106-710, 30500 Mound Road, Warren, MI 48090-9055, USA; E-Mail: xiaosong.huang@gm.com; Tel.: +1-586-986-0836; Fax: +1-586-986-0836

**Keywords:** carbon fiber, composite, light weighting, high strength, high modulus

## Abstract

This paper reviews the research and development activities conducted over the past few decades on carbon fibers. The two most important precursors in the carbon fiber industry are polyacrylonitrile (PAN) and mesophase pitch (MP). The structure and composition of the precursor affect the properties of the resultant carbon fibers significantly. Although the essential processes for carbon fiber production are similar, different precursors require different processing conditions in order to achieve improved performance. The research efforts on process optimization are discussed in this review. The review also attempts to cover the research on other precursor materials developed mainly for the purpose of cost reduction.

## 1. Introduction

Carbon fiber is defined as a fiber containing at least 92 wt % carbon, while the fiber containing at least 99 wt % carbon is usually called a graphite fiber [1]. Carbon fibers generally have excellent tensile properties, low densities, high thermal and chemical stabilities in the absence of oxidizing agents, good thermal and electrical conductivities, and excellent creep resistance. They have been extensively used in composites in the form of woven textiles, prepregs, continuous fibers/rovings, and chopped fibers. The composite parts can be produced through filament winding, tape winding, pultrusion, compression molding, vacuum bagging, liquid molding, and injection molding.

In recent years, the carbon fiber industry has been growing steadily to meet the demand from different industries such as aerospace (aircraft and space systems), military, turbine blades, construction (non-structural and structural systems), light weight cylinders and pressure vessels, off-shore tethers and drilling risers, medical, automobile, sporting goods, *etc*. [2,3,4,5,6,7]. For the automotive industry, fiber reinforced polymeric composites offer reduced weight and superior styling. Carbon fibers can find applications in body parts (doors, hoods, deck lids, front end, bumpers, *etc*.), chassis and suspension systems (e.g., leaf springs), drive shafts and so on.

The estimated global carbon fiber consumption is shown in Table 1 [2,3]. Table 2 shows the major carbon fiber manufacturers around the globe and their estimated name plate capacities [2]. A steady increase in both production and consumption in the future can be predicted. In fact, most of the carbon fiber manufacturers have plans for expansion to meet the market demand. However, the large-volume application of carbon fiber in automotive industry has been hindered due to the high fiber cost and the lack of high-speed composite fabrication techniques [4].

**Table 1:** Estimated global carbon fiber consumption [2,3].

	1999 (tons)	2004 (tons)	2006 (tons)	2008 (tons)	2010 (tons)
Aerospace	4,000	5,600	6,500	7,500	9,800
Industrial	8,100	11,400	12,800	15,600	17,500
Sporting Goods	4,500	4,900	5,900	6,700	6,900
Total	16,600	21,900	25,200	29,800	34,200

**Table 2:** Estimated name plate carbon fiber capacity in 2005 [2].

	PAN (tons)	Pitch (tons)
Toray Industries (small tow)	9,100	
Toho Tenax (Teijin) (small/large tow)	8,200	
Mitsubishi Rayon/Grafil (small tow)	4,700	
Zoltek (large tow)	3,500	
Hexcel (small tow)	2,300	
Formosa Plastics (small tow)	1,750	
Cytec Engineered Materials (small tow)	1,500	360
SGL Carbon Group/SGL Technologies (large tow)	1,500	
Mitsubishi Chemical		750
Nippon Graphite Fiber		120

The current carbon fiber market is dominated by polyacrylonitrile (PAN) carbon fibers, while the rest is pitch carbon fibers and a very small amount of rayon carbon fiber textiles. Different precursors produce carbon fibers with different properties. Although producing carbon fibers from different precursors requires different processing conditions, the essential features are very similar. Generally, carbon fibers are manufactured by a controlled pyrolysis of stabilized precursor fibers. Precursor fibers are first stabilized at about 200–400 °C in air by an oxidization process. The infusible, stabilized fibers are then subjected to a high temperature treatment at around 1,000 °C in an inert atmosphere to remove hydrogen, oxygen, nitrogen, and other non-carbon elements. This step is often called carbonization. Carbonized fibers can be further graphitized at an even higher temperature up to around 3,000 °C to achieve higher carbon content and higher Young’s modulus in the fiber direction. The properties of the resultant carbon/graphite fibers are affected by many factors such as crystallinity, crystalline distribution, molecular orientation, carbon content, and the amount of defects. Before packaging, the relatively inert surfaces of the carbon/graphite fibers are post treated to improve their adhesion to composite matrices.

In terms of final mechanical properties, carbon fibers can be roughly classified into ultra high modulus (>500 GPa), high modulus (>300 GPa), intermediate modulus (>200 GPa), low modulus (100 GPa), and high strength (>4 GPa) carbon fibers [5,6]. Carbon fibers can also be classified, based on final heat treatment temperatures, into type I (2,000 °C heat treatment), type II (1,500 °C heat treatment), and type III (1,000 °C heat treatment) [5,6]. Type II PAN carbon fibers are usually high strength carbon fibers, while most of the high modulus carbon fibers belong to type I.

Activated carbon fibers contain a large amount of open pores and are mainly used for gas absorption applications. This topic will not be covered in this review.

## 2. Structures and Properties

**Figure 1:**
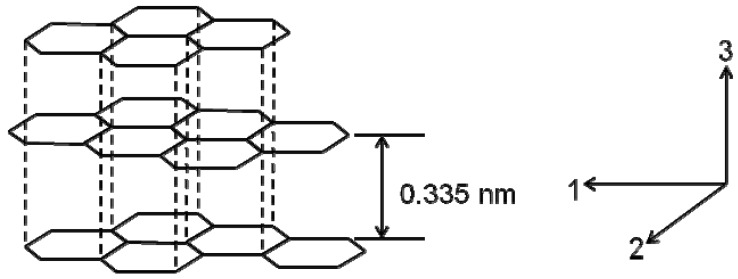
Structure of graphitic crystals and crystal directions.

The atomic structure of a carbon fiber is similar to that of graphite, consisting of carbon atom layers (graphene sheets) arranged in a regular hexagonal pattern, as shown in Figure 1. Depending upon the precursors and manufacturing processes, layer planes in carbon fibers may be either turbostratic, graphitic, or a hybrid structure. In graphitic crystalline regions, the layer planes are stacked parallel to one another in a regular fashion. The atoms in a plane are covalently bonded through *sp^2^* bonding while the interaction between the sheets is relatively weak Van der Waals forces. In a single graphitic crystal, *d*-spacing between two graphene layers (d_002_) is about 0.335 nm. Elastic constants of these single crystals have been calculated [1]. C_11_ and C_33_ are 1,060 GPa and 36.5 GPa, respectively, but C_44_ for shearing is as low as 4.5 GPa. However, the basic structural unit of many carbon fibers consists of a stack of turbostratic layers. In a turbostratic structure, the parallel graphene sheets are stacked irregularly or haphazardly folded, tilted, or split. It has been reported that the irregular stacking and the presence of *sp^3^* bonding can increase *d*-spacing to 0.344 nm [5,8]. Johnson and Watt [9] investigated the crystallite structure of a PAN carbon fiber treated to 2,500 °C and reported that the turbostratic crystallites had Lc (crystallite height) of at least 12 layer planes and La (crystallite width) of 6–12 nm. Both Lc and La tend to increase with the heat treatment temperature.

Generally, a well stacked graphitic crystalline structure can only be observed in mesophase pitch (MP) and vapor grown carbon fibers, while the turbostratic structure can be observed in carbon fibers from other precursors such as PAN. In the graphitization of stabilized PAN fibers, crystallites grow either by coalescing with adjacent crystallites or by incorporating surrounding disorganized carbons. In addition, the layer planes within the crystallites rearrange through rotating and shifting. However, the degree of these organizations is small and the graphite fibers are still turbostratic with the existence of extensive rotational misalignment of layer planes.

**Figure 2:**
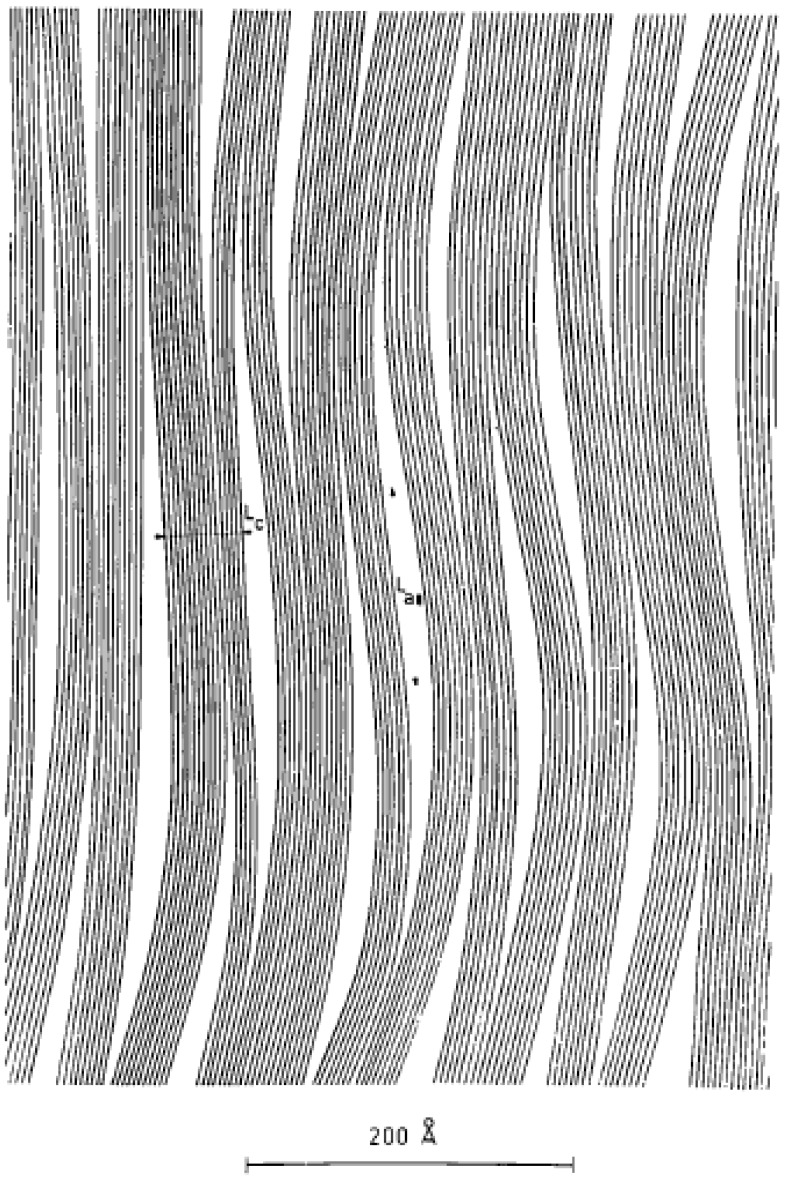
Microstructure of PAN carbon fibers [13] (Reproduced with permission from International Union of Crystallography (http://journals.iucr.org/), © 1970).

The carbon fiber microstructure depends on the precursors and processing conditions. Different models to depict the microstructures have been proposed. In Wick’s model [8,10], the graphite layers are aligned parallel to the fiber direction while stacked randomly in the transverse direction. In the transverse direction, the crystal regions (microdomains) are separated by microvoids while in the longitudinal direction, regions are separated by zones of extensive bending and twisting of the basal layers (dislocations). Watt and Johnson [11] studied PAN carbon fibers and reported a branched microfibrillar structure with most of the fibrils aligned in the fiber axial direction. The width of these fibrils is about 10 nm. Fourdeux, Ruland and Perret [12] proposed a similar wrinkled ribbon model for rayon carbon fibers. The ribbon shaped monatomic layer has an average width of 6 nm and length in the order of several hundred nm. The ribbons with different contours are stacked in parallel to form wrinkled microfibrils. Needle shaped microvoids with a diameter of about 1–2 nm are enclosed in the stacked layer planes. The sharp density transition from voids to microfibrils indicates that the wall of voids is the carbon layer surface. Pores could be formed due to the elimination of heteroatoms in heat treatment. Perret and Ruland [13] have reported that the voids have a preferred orientation in the fiber axial direction. They studied the relationship between the orientation of crystallites and Lc in PAN and rayon carbon fibers and found that the crystallites with an orientation closer to the fiber axis showed larger Lc. They also observed that ribbon like layers stacked to form microfibrils and the thickness of the microfibril parallel to the fiber axis was the largest. The proposed model is shown in Figure 2. The research by Diefendorf and Tokarsky [14] using TEM also supported the existence of a wrinkled ribbon structure in PAN carbon fibers and they proposed a 3-D model to demonstrate the microdomains.

**Figure 3:**
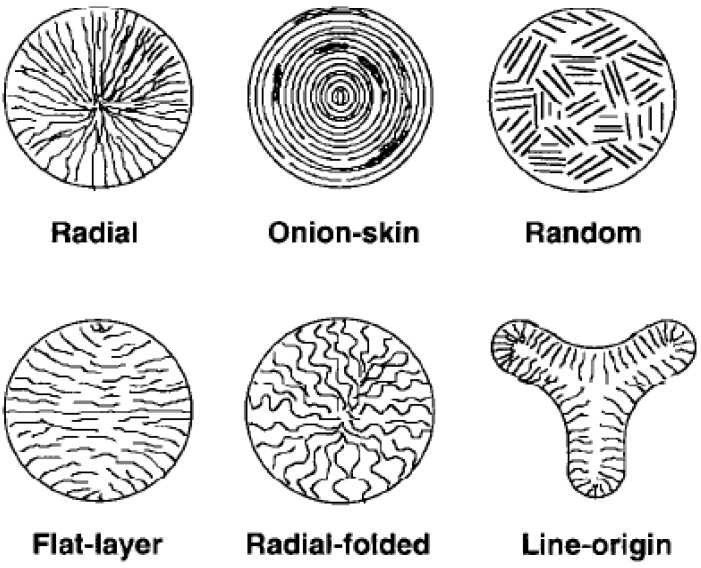
Microstructure of MP carbon fibers [15] (Reproduced with permission from Elsevier, © 1998).

Edie *et al*. [15] reported that the transverse texture of early commercial MP carbon fibers was either radial or flat-layer as shown in Figure 3. Molecular orientation in the axial direction has been created by shear when the liquid crystalline precursor flows through a capillary. Guigon and Oberlin [16] studied MP carbon fibers and observed three structures including graphite, a microporous turbostratic phase, and a phase similar to high modulus PAN carbon fibers. The three phases were distributed randomly. Fitz Gerald *et al*. [17] proposed that only two domains of a dense structure and a microporous phase existed. The microdomains were formed during spinning and the organization occurred within domains during the subsequent heat treatment.

Some carbon fibers also exhibit a sheath-core structure. Diefendorf and Tokarsky [14] reported that ribbons in fiber core had preferred radial distribution and the layers in the surface tended to be circumferential forming an onion like skin. Huang and Young [18] confirmed the existence of the sheath-core structure in PAN carbon fibers using Raman spectroscopy. The two regions in the precursor fibers are responsible for the formation of the sheath-core structure in the resultant carbon fibers.

Kobets and Deev [19] observed different PAN and hydrated cellulose carbon fibers and their research supported a microcomposite structure. The conclusion was obtained based on SEM observations and the fiber behaviors at high temperatures. In this model, quasi-amorphous carbon matrix is reinforced by orientated carbon fibrils of about 150–400 nm. The fibrils are composed of microfibrils of 50–100 nm and crystallite packets of up to 20 nm. The shear and compressive strength, and thermal stability depend on the micromatrix surrounding the fibrils. An isotropic carbon layer up to 10 nm was also observed in the fibers investigated. Based upon the model and the mechanical tests, they proposed that the fiber strength could be increased by decreasing the size of reinforcing elements and decreasing stress concentration on the fiber surface and at stochastic defects.

It is well known that microstructure affects fiber properties. Due to the high content of delocalized π electrons and the parallel alignment of graphene layers along the fiber axis, carbon fibers show good thermal and electric conductivities in the fiber direction. Their coefficient of thermal conductivity is in the range of 21–125 W/mK, which is close to that of metals [20]. In the case of high modulus MP carbon fibers, thermal conductivity can be more than 500 W/mK at room temperature. The electric conductivity of graphitized carbon fibers is also close to that of metals [20]. High modulus MP carbon fibers with a high crystal orientation generally show a higher electric conductivity than PAN carbon fibers.

One thing to be noted is that carbon fibers have a small but negative coefficient of linear thermal expansion (CLTE) of –0.5~–2.0 × 10^-6^/K in the fiber axial direction. The negative CLTE is due to the high crystalline alignment in carbon fibers. It has been reported that with the increase of Young’s modulus in the fiber direction, the absolute value of CLTE increased [21]. The negative CLTE can cause residual stresses at the interface when the composites are subject to temperature change.

The high modulus of carbon fibers comes from the high crystallinity and the well alignment of crystals in the fiber direction, while the strength of carbon fibers is primarily affected by the defects and crystalline morphologies in fibers [5]. MP carbon fibers usually show higher elastic modulus but lower tensile strength than PAN carbon fibers [22]. The mesophase (liquid crystalline) spinning dopes for MP carbon fibers contain both anisotropic and isotropic phases. The shear force in the spinning process aligns the anisotropic phase to form a well orientated structure with a relatively large crystal size. In contrast, amorphous PAN dope is spun into precursor fibers through solution spinning and the molecular alignment is achieved by the shear in spinning as well as the fiber drawdown in the following processes. Due to the structural difference in precursors, the crystallites in MP carbon fibers are larger in size and tend to be more graphitic with inter lamellar spacing in the range of 0.337–0.340 nm, compared to PAN carbon fibers which contain mainly turbostratic crystals [1,22]. Therefore, MP carbon fibers usually have higher Young’s modulus and better thermal and electrical conductivity in the fiber direction [5]. However, larger crystallites also result in higher stress concentrations on grain boundaries. In addition, the extended graphitic structure in MP carbon fibers makes the fiber more sensitive to defects [4]. Thus, PAN carbon fibers with smaller turbostratic crystallites generally offer higher tensile strength [22,23]. Increasing heat treatment temperature to develop a larger and better aligned graphitic structure can improve fiber Young’s modulus while removing flaws has the potential to improve fiber strength. Reynolds and Sharp [23] proposed that instead of flaws, tensile failure started from disoriented crystals by a localized shear mode since crystallites were weakest in shear on basal planes. Johnson and Thorne [24] have tried using oxidation to remove surface flaws for improved fiber strength. After treating carbon fiber in air for about 10 min, they observed an 80% increase. Thorne [25] improved the fiber tensile strength by about 70% by heat treating carbon fibers in a mixture of carbon dioxide and acetylene at about 700 °C. The deposition of carbon on the fiber surface healed flaws contributing to the improved properties. Endo [26] observed that the MP carbon fibers with higher tensile strength showed more turbostratic structure. The folded turbostratic structure has more tortuosity, which hinders crack propagation.

Carbon fibers have low compressive strength, partially due to the weak Van der Waals force between graphene layers and their fibrillar structure. MP carbon fibers usually have lower compressive strength than PAN carbon fibers [5]. Dobb *et al*. [1,27] investigated the compressive failure of carbon fibers by examining the SEM images after the recoil test. They observed that high modulus PAN carbon fibers buckled on compression. The failure initiated from the side on tension while the other side on compression formed kink bands. For high modulus MP carbon fibers, due to the better orientation of the carbon layers, the compressive failure was through a shear mechanism and a kink band at 45° to the fiber axis [5]. Hahn and Sohi [28] prepared unidirectional composites using T-300 and T-700 PAN carbon fibers from Toray, and P-75 MP carbon fiber from Amoco. They concluded that the PAN fibers failed by microbuckling while MP carbon fiber formed a shear band.

Fiber torsional behaviors are also related to the microstructure. Compared with PAN carbon fibers, MP carbon fibers show lower torsional modulus because their radial structure facilitates shear between layers. The highly random distribution of layers in the fiber cross-section contributes to the higher torsional modulus of PAN carbon fibers.

## 3. PAN Carbon Fibers

Polyacrylonitrile (PAN), containing 68% carbon, is currently the most widely used precursor for carbon fibers.

### 3.1. Precursor Fiber Preparation

PAN can be polymerized from acrylonitrile (AN) by commonly used initiators, such as peroxides and azo compounds, through the addition polymerization process. The process can be either solution polymerization or suspension polymerization. The solution polymerization is preferred to be conducted in a PAN solvent so that the produced PAN solution can be used as a fiber spinning dope directly, once the unreacted monomers are removed. This eliminates the PAN drying and redissolving processes. The solvent needs to have a low chain transfer coefficient in order to produce PAN with increased molecular weights. The most commonly used solvents are dimethyl sulfoxide, ZnCl_2_ and NaSCN [29]. But the molecular weight of the PAN/PAN copolymer produced by this process is usually low. An alternative process is suspension polymerization in which linear polymers with higher molecular weights can be obtained. The molecular weight of PAN/PAN copolymer for spinning is usually in the range of 70 k–260 k and the polydispersity index is preferred to be 1.5–3.0 [5]. Either a batch or a continuous process is used for PAN polymerization. Reproducible PAN polymer can be obtained with a batch process, but the produced PAN usually has a relatively wide molecular weight distribution [29]. A continuous process is suitable for large scale production but it is difficult to control the product quality [29].

Linear PAN has polar nitrile groups in the molecules, resulting in strong intermolecular interactions. Because of this strong intermolecular interaction, PAN has a high melting point and, therefore, it tends to degrade before the temperature reaches its melting point. Solution spinning appears to be the only proper choice for PAN precursor fiber spinning. PAN copolymers are usually used as precursors in various industries. Typically, an approximate 5 mol % of comonomers (such as methyl acrylate and vinyl acetate) is incorporated as internal plasticizers to reduce the intermolecular interaction to improve the solubility of PAN polymer and the processibility of PAN precursor fibers [5]. The incorporation of a comonomer can also improve the carbon fiber mechanical properties due to the increased molecular orientation in precursor fibers and the resultant carbon fibers. Some comonomers, especially those with acidic groups (like acrylic acid or itaconic acid) or acrylamide, facilitate the cyclization reaction in the stabilization step and, for that purpose, 0.4–1 mol % is usually incorporated in the copolymer [5,6,30]. Henrici-Olive and Olive [31] used vinyl bromide as the comonomer. They reported that the dehydrobromination would readily occur and the released HBr acted as an initiator for stabilization reactions.

Traditional wet spinning has been widely used to produce PAN precursor fibers, although newly developed dry jet wet spinning has shown to be able to spin a dope with higher polymer concentrations and produce carbon fibers with better mechanical properties. In wet spinning, PAN is first dissolved into a highly polar solvent, such as dimethyl formamide, dimethyl acetamide, sodium thiocyanate or their mixtures, to form a solution of 10–30 wt % [5]. PAN solution is then filtered and extruded. The extruded PAN goes through a coagulation bath consisting of a PAN solvent and a non-solvent and fibers are consolidated when the solvent diffuses away from the precursor. Fiber bundles are under stress in the coagulation bath to achieve the molecular alignment. The higher concentration of the non-solvent and the higher temperature of the coagulation bath, the higher is the coagulation rate. In wet spinning process, a low coagulation rate is preferred to avoid defects both inside of the fibers and on the fiber surfaces. A low coagulation rate can also suppress the formation of the unpreferred skin-core structure [5]. With a high concentration of solvent in the coagulation bath, fibers in a gel state is formed. Orientation can be easily achieved in this state. The PAN precursors pass though several baths with different temperatures and compositions to allow better molecular orientation in the precursor fibers. The residence time in the bath can be as short as around 10 sec [5]. Mitsubishi Rayon Co. [32] reported that an ultrasonic wave assisted coagulation process improved the tensile strength of the obtained PAN precursor fibers. The coagulated fibers are then washed and further stretched in steam to remove the excess solvent in the fibers and increase the molecular orientation. Fibers can be drawn at a higher temperature of 130–150 °C by using glycerol as a drawing medium [5]. Further increase in tensile properties is observed as the orientation increases through the high temperature drawing. Wet spun precursor fibers usually show a microfibrillar structure with the fibrils orienting along the fiber axis. Fibers coming out of the orifice are round due to the slow and uniform diffusion of solvent from precursor fibers, but collapsing in the coagulation bath leads to different fiber cross-sectional shapes and the effect is pronounced if the concentration of the spinning solution is low. Knudsen [33] has reported that spin bath temperature was one of the most important factors that affected the cross-sectional shape of a precursor fiber. The temperature of 10 °C produced bean shaped fibers, while the temperature of 50 °C produced round fibers. The amount of voids, existing between microfibrils, was reported to decrease when lowering the coagulation temperature to 10 °C [33].

The drawback of solution spinning (including wet, dry, and dry jet wet spinning) is the added cost for solution recovery. A melt-assisted spinning process has then been developed to reduce the processing cost [34,35,36]. In the melt-assisted spinning process, a diluent, functioning as a plasticizer, is used to reduce the interaction between PAN molecules by decoupling the nitrile-nitrile association. Thus, the melting point of plasticized PAN is reduced to an appropriate range for melt spinning [34,35,36]. Grove *et al*. [37] have obtained melt spun PAN fibers using water as a plasticizer. They observed the existence of surface defects and internal voids and concluded these defects resulted in the relatively low mechanical properties of 2.5 GPa tensile strength and 173–214 GPa tensile modulus. BASF Structural Materials, Inc. [34,35,36] patented a process for melt assisted spinning of PAN fibers. The precursor was a PAN solution with a mixture of water, acetonitrile and an optional monohydroxy alkanol/nitroalkane as the diluent. Fibers were extruded into a pressure vessel to avoid the instant release of volatiles which can cause defects. The initially stretched precursor fibers passed through a heat treatment zone to release residual acetonitrile and water. Further stretching and high temperature carbonization were followed. The carbonized fibers showed higher tensile strength of about 3.6 GPa and modulus of about 233 GPa with elongation of 1.54%. This process significantly reduces the amount of hazardous solvents used for the spinning dope.

Researchers have also been putting efforts on developing solvent free melt-spinnable PAN precursors to reduce carbon fiber cost caused by the solvent recovery and to achieve higher spinning rate/output. It has been noticed by some researchers [38,39] that copolymers containing 85–90 mol % AN melt below their degradation temperature and have melt viscosity in the processible range of 100–1,000 Pa·s at 220 °C. However, the incorporation of a high content of comonomer may hinder the cyclization of the nitrile groups in the stabilization process, which lowers the properties of precursor fibers and the resultant carbon fibers. In addition, the reduced melt temperatures of PAN copolymers or terpolymers may require a reduced stabilization temperature resulting in a prolonged stabilization process.

To accelerate fiber stabilization, fibers can be crosslinked through electron beam irradiation or UV light before stabilization [38,39,40]. A photo-crosslinkable and melt processible PAN terpolymer precursor was prepared and investigated by McGrath *et al*. [38]. The mole ratio of AN/methyl acrylate/acryloyl benzophenone was 85/14/1. The terpolymer was UV crosslinked before stabilization for reduced processing time. With an average diameter of 7 µm, the resultant carbon fibers showed tensile strength up to 600 MPa and modulus up to 126 GPa.

Imai *et al*. [41] patented a PAN spinning dope containing ZnCl_2_ and HCl, and a process for spinning such a dope. PAN was solution polymerized in a concentrated ZnCl_2_ aqueous solution at a pH less than 2 adjusted by HCl. The concentrated aqueous ZnCl_2_ is a good solvent for PAN and the low content of HCl has shown a significant effect on preventing the precursor fiber agglutination. The polymer solution in the presence of ZnCl_2_ and HCl was spun into precursor fibers with ZnCl_2_ aqueous solution as a coagulant. The spun fibers were rinsed to lower the Zn content, applied with an oily processing agent and dried. The carbon fiber produced from this method showed tensile strength of more than 5.4 GPa. Similarly, in a patent to Ohsaki, Imai and Miyahara [42], a high strength carbon fiber was produced from precursors produced by extruding an aqueous PAN/ZnCl_2_ solution. The precursor fiber was prepared by extruding a 2–7 wt % PAN solution with aqueous ZnCl_2_ as the solvent into a coagulating bath containing less concentrated ZnCl_2_ at a specified draft ratio. Conventional washing and drying was followed with a total stretching ratio of 10–20 folds. The carbon fibers produced this way showed circular cross-section with circumferential ruggedness extending in the fiber axial direction. Maximum strength of more than 5 GPa has been achieved.

Some researchers have started the evaluation of isotactic PAN as a carbon fiber precursor since isotactic PAN shows different cyclization behaviors from the conventional atactic PAN [43,44]. Hirotaka and Hiroaki [43] patented a process for preparing highly isotactic PAN copolymers as carbon fiber precursors through solid phase-polymerization. Teijin Limited [43] patented a potential PAN carbon fiber precursor that was comprised of at least 50 wt % of AN wherein the isotactic triad proportion within the AN structural chain was at least 35 mol %. This precursor polymer allowed finishing flame retarding treatment at a lower temperature, and/or in a shorter time. No data on the carbon fiber fabrication from the isotactic PAN has been reported. However, other researchers reported that the stereoregulatity of PAN was not so important since the isotope exchange could occur rapidly at elevated temperatures [45].

In seeking low cost PAN fibers, Hexcel Corp. (Sandy, UT, U.S.A.) has developed methods of using a textile-grade PAN as the precursor for automotive industry with targeted modulus and strength of 172 GPa and 2.7 GPa, respectively [46,47,48]. The conventional precursor grade PAN and textile grade PAN are different in several ways. Precursor PAN has smaller diameter for the ease of stabilization and heat treatment compared to textile PAN. Exothermic reactions in stabilization are controlled better with smaller sized fibers. The molecular weight and molecular weight distribution of precursor PAN are usually well controlled for the optimal spinning and heat treatment. In addition, precursor PAN has higher purity and higher AN content (normally >90%) for improved properties of precursor fibers and resultant carbon fibers [4]. Virgin textile PAN is not suitable for carbon fiber production due to the uncontrollable oxidation or extremely long time for stabilization, while the textile grade PAN chemically treated with hydrogen peroxide in an alkaline medium can be stabilized and carbonized using available industrial facilities and infrastructures [46,47,48].

To avoid adhered, fluffy, or broken fibers, precursor fibers are coated with finishing oil before heat treatment or sometimes before the drying of the precursor fibers. Traditionally, polyoxyethylene, silicon oils, and fatty acid derivatives are used as the finishing oil. Shiromoto *et al*. [49] patented an oiling agent with good heat resistance. It comprises a neopentyl alcohol derivative with an optional 10%–80% of a modified polysiloxane. In another patent [50], a finish containing the reaction product of a saturated aliphatic dicarboxylic acid and a monoalkyl ester of an alkylene oxide adduct of bisphenol A was applied. It eliminated fused or broken precursor fibers effectively.

### 3.2. Oxidization/Stabilization

**Figure 4:**
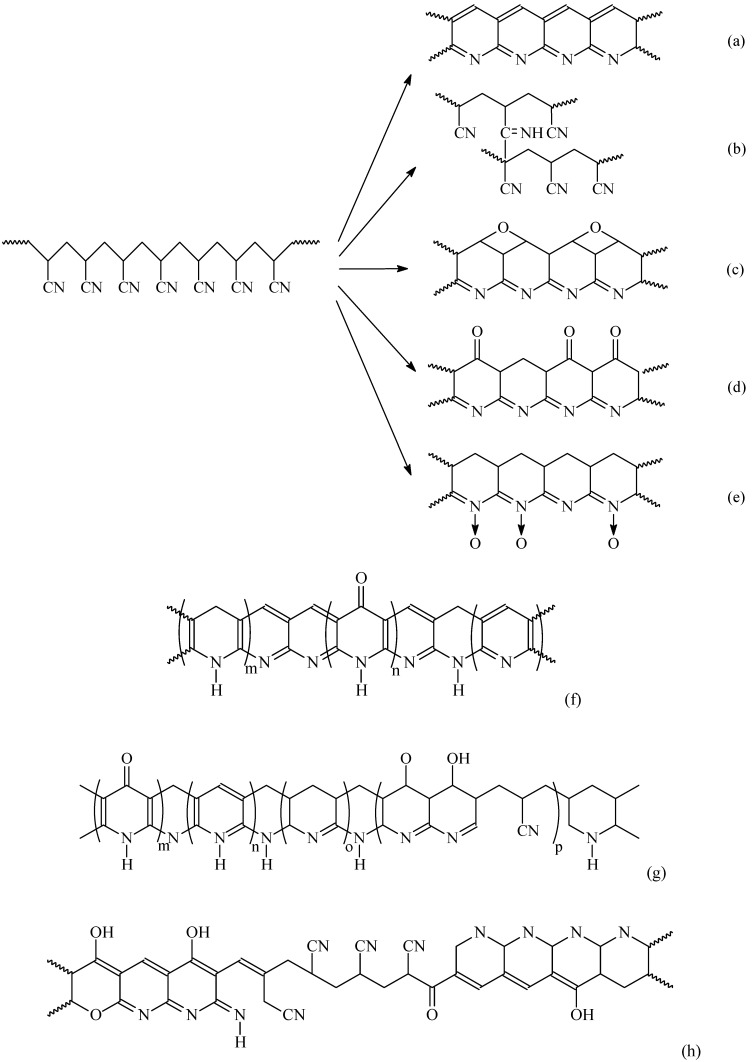
Oxidization of PAN.

In the stabilization step, the linear PAN molecules are first converted to a cyclic structure. However, cyclization is a very complicated process and there are still differences of opinion on the reaction mechanisms. Different models have been proposed for the cyclization of PAN molecules as shown in Figure 4. Houtz [51] proposed a cyclized and dehydrated structure shown in Figure 4 (a). This structure is often cited as the product of low temperature stabilization. Schurz and co-workers [52] stated that an azomethine crosslink could be formed as shown in Figure 4 (b). Since both structures contain no oxygen, Standage and Matkowshi [53] proposed an oxidized structure as in Figure 4 (c), Watt [20] proposed a ladder structure as in Figure 4 (d), and Friedlander *et al*. [7] showed a nitrone structure as in Figure 4 (e). Some researchers have tried to include different structures in the oxidized molecules. For example, Clarke *et al*. [54,55] proposed a structure as shown in Figure 4 (f), Bailey and Clarke [56] suggested a structure as in Figure 4 (g), and Goodhew *et al*. [57] proposed a structure as in Figure 4 (h). Unreacted nitrile groups are present in (g) and (h) due to the random initiation sites and the atactic nature of PAN. The orientation in the pyrolysis step is maintained by the strong intermolecular hydrogen bonding and the rigidity of the ladder structure.

FTIR results have supported an intramolecular reaction according to some researchers [6,8], but the reactions cannot be explained from the aspect of stereochemistry or elemental analysis. Intramolecular reactions are more stereospecific than intermolecular reactions. Due to the repulsion between nitrile groups, the PAN molecules are not in the form of extended chains, but a rod-like helix structure with CN groups positioning at different angles guided by the intramolecular repulsion and intermolecular attraction [58]. Thus, intermolecular reactions are preferred. However, the research conducted by Granster *et al*. [59] suggested that the adjacent nitrile groups were in attraction and the molecular structure was between planar zig-zag and helix structures. Gupta and Harrison [60] studied the PAN copolymer and concluded that intramolecular cyclization reactions within the rod-like helix dominated in the early stabilization (roughly below 300 °C), while intermolecular cross-linking took place at higher temperature and/or in the presence of oxygen. They also stated that the amorphous phase in PAN copolymer precursor fibers was initiated at about 175 °C followed by the breakdown and intramolecular cyclization of the crystalline phase at higher temperatures.

Stabilization (cyclization and oxidization) is an exothermal reaction, thus heating pattern needs to be well controlled. A high heating rate results in a large amount of heat released in a short time, which can reduce carbon yield and introduce defects in precursor fibers. Lower heating rate is preferred also because it will allow oxygen to diffuse into the core of the precursor fibers to finish the stabilization completely. The cyclization of PAN and PAN copolymers was investigated by Fitzer and Muller [61]. The amount of heat and the temperature range in which the heat was produced were heavily dependent on the composition of the polymers. It was concluded that the cyclization of the copolymers was slowly initiated by ions while the cyclization of PAN polymer was initiated mainly by free radicals [61,62]. Therefore, the incorporation of acidic groups reduces exotherm and the onset cyclization temperature. It was reported that the incorporation of 2% methacrylic acid in PAN molecules together with a treatment in an acidic solution reduced the stabilization time from hours to 25 min [63]. The treatment in acid was conducted by stretching the precursor fibers in a hot acidic aqueous solution at a pH below 3.5 and temperatures above 90 °C [63]. Fitzer *et al*. [62] has studied the stabilization of PAN with 6% methyl acrylate and 1% itaconic acid by controlling precursor fibers at constant length while heating at different heating rates with the same onset temperature of 230 °C. To achieve optimal carbon fiber properties, the recommended treating condition was a heating rate of 1 °C/min and a treating time of 40 min. After carbonization to 1,500 °C, carbon fibers with tensile strength of 3.65 GPa were obtained. Mitsubishi Rayon Co. [64] disclosed an AN-based precursor containing carboxylic acid groups and acrylamide units for increasing the stabilization rate and sulfate/sulfonic groups for controlling the denseness of the precursor fibers. Counter ions were preferred to be protons and/or ammonium ions to avoid the reduction in carbon fiber properties caused by alkali metals. The carbon fibers formed from this precursor showed a tensile strength of 5.4 GPa and a modulus of 268 GPa. Residues from the spinning solution can also behave as an initiator for nucleophilic cyclization reactions to reduce stabilization time. Potter and Scott [65] proposed that the sodium thiocyanate residue from the spinning solution accelerated the nitrile cyclization reaction.

Different media have been tried to control the exotherm and moderate the released heat to increase the stabilization rate. The presence of oxygen accelerates the initiation of cyclization and the formation of crosslinking among linear polymer chains. It has been reported that the optimum oxygen uptake for improved carbon yield and mechanical properties is 8%–10% [6,66]. There are two controlling processes in this step. When the rate of ladder structure formation is slower than the diffusion of oxygen into the fibers, it is chemistry controlled. Or else, it can be diffusion controlled [20]. In most cases, stabilization rate is controlled by the diffusion of oxygen into the precursor fibers. The kinetics can be changed by many factors, such as precursor compositions, fiber structures, fiber diameters, stabilizing temperature, and the environment.

Researchers have also reported that oxidization in SO_2_, HCl or Br_2_ resulted in more crosslinks and thus higher carbon yield [67,68]. Hydroxylamine solution [69], aminophenoquinones [70], aminosiloxanes [71], and primary amine or quaternary ammonium salts [72] have been used as precursor fiber impregnation bath or added in the spinning dope directly for controlling exotherm and improving stabilization. The application of persulphate [73], cobalt salt [74], hydrogen peroxide [75], potassium permanganate [76], dibutyltindimethoxide [77], aqueous guanidine carbonate [78] and hydrazine hydrate [79] has also shown to be able to modify the structure of the stabilized precursor and increase the stabilization rate. Not only can they improve the stabilization reactions, some of them can also heal the fiber surface defects through the deposition on the fiber surface in the high temperature heat treatment.

Researchers at Oak Ridge National Lab (ORNL) believe that the plasma processing can enhance oxygen diffusion and oxidation reactions, and developed a low-temperature plasma oxidation process to significantly reduce the retention time. To avoid the filament adhesion and tow rigidity caused by exothermic heating, precursor fibers need to be lightly stabilized before being exposed to plasma oxidization at atmospheric pressure. They have evaluated different processes including electron-beam, thermochemical and ultraviolet treatments. The thermochemical based plasma process was reported to be able to reduce the retention time from 100–120 min in conventional process to less than 35 minutes [46,80].

### 3.3. Carbonization and Graphitization

The stabilized fibers are then heated in an inert environment (N_2_) to more than 1,500 °C under slight tension for a period of a few minutes depending on the fiber diameter, composition, and morphology. The importance of stretching in this step is still under debate. The fiber diameter is reduced with the removal of the non-carbon elements. At the early stages of carbonization, crosslinking reactions take place in the oxidized PAN. The cyclized structure starts to link up in the lateral direction by dehydration and denitrogenation. A planar structure can be formed with the basal planes oriented along the fiber axis. These fibers are generally called “high strength” fibers. The strength of a carbon fiber is observed to increase with the carbonization temperature and the maximum strength is observed at around 1,500 °C. Further increase in temperature results in increased modulus but reduced tensile strength [6]. Too fast a carbonization rate introduces defects in carbon fibers, while low carbonization rate causes the loss of too much nitrogen at the early stages of carbonization, certain amount of which is preferred to achieve high strength carbon fibers.

**Figure 5:**
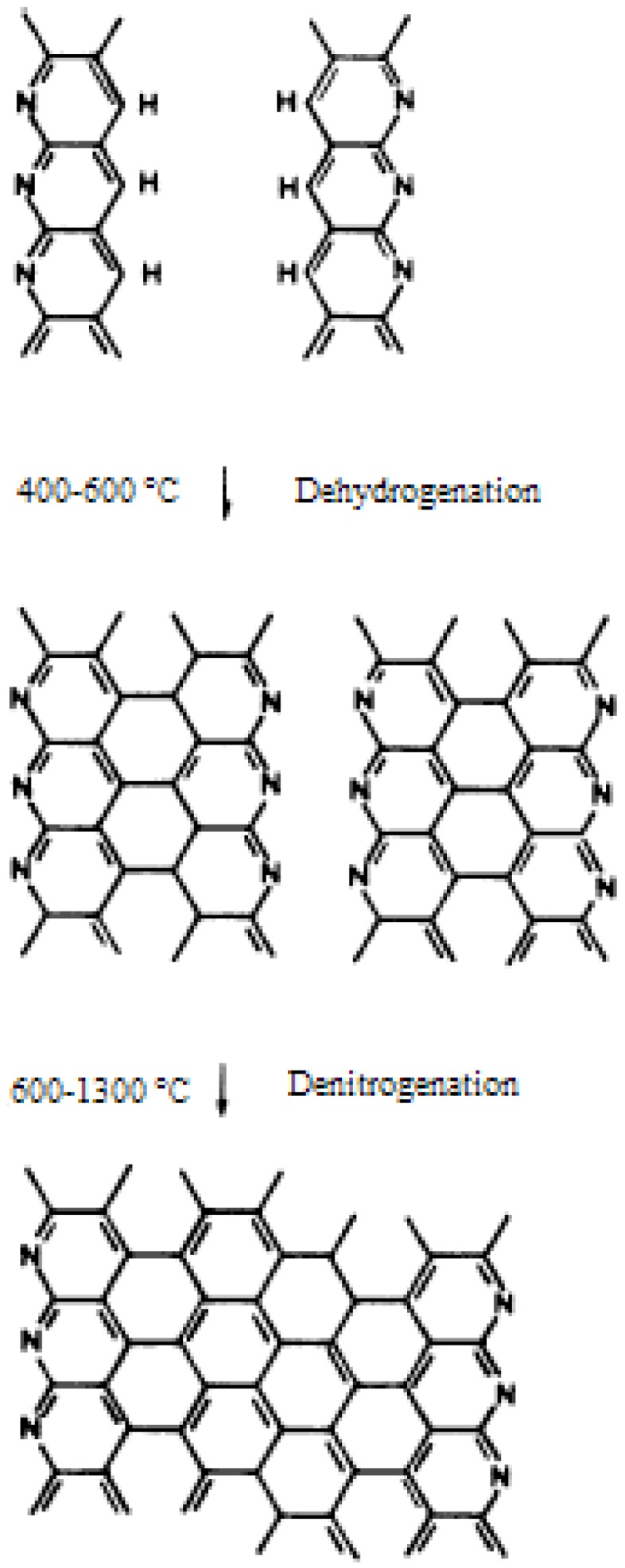
Schematic formation of the graphite structure [57] (Reproduced with permission from Elsevier, © 1975).

Watt [20] studied the released gases from both stabilized and unstabilized PAN fibers in vacuo at different pyrolysis temperatures. Two release peaks were observed. The HCN and NH_3_ evolved at temperatures up to 450 °C were from an unladdered structure while in the range of 450–900 °C were from an laddered structure resulting in polymer crosslinking. The release of N_2_ was observed starting from 700 °C. Goodhew *et al*. [57] suggested that intermolecular dehydrogenation took place between 400 °C and 600 °C, while denitrogenation took place at higher temperatures. The schematic of the development of the graphitic structure is shown in Figure 5. Dehydrogenation joined ladder molecules forming graphite-like ribbons, whereas, denitrogenation was responsible for the growth of ribbons to form sheet like structures. The sheets could further grow at higher temperatures with the release of N_2_. Deurberque and Oberlin [68] conducted research at a low pyrolysis rate of 4 °C/min. They concluded that to produce high performance carbon fibers, a high nitrogen content (large N/C ratio) should be retained when carbon skeleton was being rearranged till the later stages of carbonization. Molecular ordering is easy when molecules remain flexible in the presence of nitrogen. The nitrogen in aromatic rings can form bonding between layer planes with the release of N_2_, thus increasing the compactibility and resulting in improved tensile strength [56]. The graphene sheets contain defects and can fold enclosing voids that are oriented in the fiber direction. Voids and defects tend to decrease at higher treatment temperatures by joining the adjacent graphite-like layers and further aligning them in the fiber direction.

The produced carbon fibers can be heated to even higher temperatures of more than 2,000 °C (graphitization) to achieve a higher modulus. Increasing the heat treatment temperature is responsible for the growth of the ordered structure in both thickness and area, the increased crystalline orientation in the fiber direction, and the reduction of the interlayer spacing and the void content. The decrease in tensile strength is explained by the increased local defects as discussed earlier. Argon is usually used in this step since N_2_ can react with carbon at such high temperatures. It has been reported that the diffusion of boron into the heat treated fibers can increase the fiber Young’s modulus [8]. The fibers were treated in an atmosphere containing boron to get boron atoms infused into the fibers. Boron atom has a small radius and can fit into the graphene lattice. Boron was suggested to be able to improve the process of recrystallization and increase the shear modulus by solid solution hardening [81].

ORNL researchers evaluated a microwave generated plasma process to reduce the carbonization time and the energy consumed in this step [46,82]. The residence time was reduced to approximately 1/3 of the conventional residence time by this process while acceptable mechanical properties were still obtained. Sung *et al*. [83] applied a high magnetic field to assist the carbonization of the stabilized PAN precursor. A magnetic field of 5 T was imposed parallel to the fiber axis at 1,172 °C and the resultant carbon fibers were graphitized at 2,000 °C without the application of any magnetic field. Due to the reduced surface defects, the tensile strength of these treated fibers was increased by 14% compared with those without the magnetic treatment. Prior to winding the continuous filaments on bobbins, the surface of carbon fibers is usually electrochemically treated and sized to improve handling properties and adhesion to the matrix resin.

## 4. Pitch Carbon Fibers

Natural pitch is produced by the destructive distillation of petroleum and coal, while synthetic pitch is produced by the pyrolysis of synthetic polymers. Pitch can contain more than 80% carbon. The composition of a pitch varies with the source tar and the processing conditions. Coal pitch is generally more aromatic than petroleum pitch. Smith *et al*. [84] has reported that 2/3 of the compounds in coal tar pitch were aromatic and the rest were heterocyclic. However, coal pitch often has a high carbon particle content (solid content), which causes filament breakage during extrusion and thermal treatment. Therefore, petroleum pitch is preferred for carbon fiber production. The commercial pitch contains aromatic compounds with molecular weights in the range of 400–600 [85]. The use of synthetic pitch has attracted more interests recently since it has a higher purity and the stabilization can occur at a faster rate at a given temperature.

Otani [86] reported the production of carbon fibers from a polyvinyl chloride (PVC) pitch in as early as 1965. The pitch was produced by pyrolyzing PVC at about 400 °C in nitrogen. The melt spun fibers were oxidized with ozone below 70 °C or in air below 260 °C and then carbonized at temperatures up to 500–1,350 °C in nitrogen. Due to the lack of crystallite orientation, the carbon fiber had Young’s modulus of about 49 GPa and tensile stress of about 1.8 GPa. Otani *et al*. [87] then reported the production of carbon fibers spun from petroleum asphalt and coal-tar pitch that were treated by bubbling nitrogen gas through them at about 380 °C and then vacuumed at the same temperature. The stabilized and carbonized fibers from this petroleum pitch showed similar properties as those produced from the PVC pitch.

Pitch as a precursor has the advantage of lower material cost, higher char yield, and higher degree of orientation compared with PAN. The graphitic structure also gives pitch based carbon fibers higher elastic modulus and higher thermal and electrical conductivity along the fiber direction [4]. However, the processing cost (mainly from pitch purification, mesophase formation and fiber spinning) to achieve high performance carbon fibers is higher. Pitch from petroleum and coal tar is isotropic. By evaporating low molecular weight fractions, isotropic pitch can be melt spun into low cost general-purpose (low strength and low modulus) carbon fibers. To produce high performance fibers, an expensive hot stretching process (explained in the following section) needs to be applied. A more common way to produce high performance carbon fibers from pitch is to use an anisotropic pitch, such as mesophase pitch.

Both isotropic and mesophase pitches are melt spinnable. Prior to fiber spinning, particulates are removed from the pitch. There is no need to hold the precursor fibers under a strong tension in the process of the stabilization and carbonization. The mesophase orients itself along the fiber axis direction during the precursor fiber spinning.

### 4.1. Mesophase Preparation and Precursor Fiber Spinning

Mesophase pitch contains an appreciable amount of anisotropic phase or liquid crystalline phase and an isotropic phase. It can be obtained by heating petroleum or coal tar pitch at 350–500 °C in an inert atmosphere. Depending on the heating temperature, it takes days to hours to obtain the desired amount of mesophase. Free radical polymerization and the evaporation of smaller molecules are responsible for the increased molecular weight at this stage [1]. The produced two phase mesophase pitch usually has a wide molecular weight distribution.

In the initial state of mesophase formation, anisotropic spheres with layers of oriented molecules aligned in the same direction are formed in the pitch when heated in an inert atmosphere. The size of the spheres grows when heat is continually applied and the spheres coalesce. The size of the domains of aligned molecules depends on the viscosity, the rate of viscosity increase, and the pitch composition. At a certain point, long range anisotropy can be achieved while the dope still has a melt spinnable viscosity (roughly 10–200 poises). A pitch with a high mesophase content requires a high spinning temperature. However, at high temperatures, mesophase can be decomposed or cross-linked in a short time in the presence of air, which significantly reduces the filament handling time. In addition, the process to improve the mesophase content or softening temperature can introduce solid particles in pitch, which reduces the pitch spinnability. On the other hand, a certain amount of mesophase is required to increase the softening temperature of the mesophase pitch, thus a higher oxidization temperature can be applied. Therefore, the molecular weight and mesophase content should be optimized for melt spinning. Pyridine or quinoline insoluble contents are usually used to characterize the mesophase content. Singer [89] has proposed that the preferred mesophase content for melt spinning is about 55% to 65%. He also concluded that homogeneous bulk mesophase with large coalesced domains in excess of two hundred microns in size, formed under quiescent conditions, were suitable for pitch fiber spinning. The infusible solids should be filtered to less than 1 wt % before producing mesophase. The existence of infusible contents hindered the formation of bulky uniform mesophase. Lewis [89] patented a faster process of producing mesophase using a reduced pressure of less than 30 mm Hg together with agitation. The reduced pressure facilitated the removal of the volatile low molecular weight fractions. Agitation has shown to produce a homogeneous emulsion of low molecular weight mesophase in an isotropic phase. As a result, a mesophase pitch having a mesophase content between 40% and 90% was prepared at a rate more than twice as fast as the above quiescent heating process.

Due to differences in viscosities and densities between the anisotropic and isotropic phases, the spinning of mesophase pitch is not very consistent. Chwastiak and Lewis [90] have reported that the process for mesophase pitch preparation could be modified by sparging an inert gas to remove volatile compounds in pitch while being agitated. The produced single-phase, low molecular weight mesophase (90% having molecular weight of less than 1,500) showed better spinnability. The conversion from isotropic pitch to mesophase pitch has also been conducted by sparging an oxidative gas through the pitch [91]. The sparging gas was nitrogen containing 0.1–2 vol % oxygen. Compared with inert gas sparging, the processing time to form 100% mesophase was reduced.

Supercritical extraction is a promising pitch-separation technique, which can remove solid impurities more efficiently and separate fractions with a narrower molecular weight distribution [4]. A process of extracting a coal tar pitch with a supercritical gas mixture of propane, toluene and benzene has been patented [92]. The obtained pitch solution, which was free of quinoline-insoluble components, was heated under a non-oxidizing atmosphere to prepare a mesophase pitch containing 40%–65% anisotrophy by volume. Further extraction of the insoluble components resulted in a mesophase pitch, which contained at least 75% anisotropy with a mean molecular weight of 900–1,200 and a melting point of 330–360 °C. Thies *et al*. [93] used a continuous-flow apparatus to fractionate a heat-soaked isotropic petroleum pitch with supercritical toluene under different conditions. 100% melt spinnable mesophase has been achieved at 340 °C and 70 bar with a solvent/feed ratio of 3:1. The carbonized fibers with a diameter of about 9 μm showed a tensile strength of around 3.3 GPa and a modulus of around 820 GPa. The mesophase yield was not reported.

Diefendorf and Riggs [94] have used solvent extraction to remove small molecules to form a neomesophase. Isotropic pitch was treated with solvents such as benzene or toluene preferably at an ambient temperature. The insoluble portion could be converted to an optically anisotropic pitch containing at least 75% of a highly oriented pseudo-crystalline neomesophase in less than 10 minutes. It is termed as neomesophase because the highly oriented, optically anisotropic pitch material has a substantial solubility in pyridine and quinoline. The mesophase formation process was significantly shortened but the removal of organic solvents added additional costs, and the final neomesophase yield was very low. The neomesophase pitch has lower softening point, thus can be processed at a lower temperature with less tendency to form coke in extrusion and stabilization. Angier and Barnum [95] patented a process of producing neomesophase with an increased yield. A typical graphitizable isotropic pitch was heated at 350–450 °C and then cooled before the spherules of liquid crystals begun to form in the isotropic pitch. The produced isotropic pitch was extracted with toluene or heptane to form a neomesophase former. The neomesophase former was then converted into neomesophase through a heating process. However, the final neomesophase yield was still relatively low, with a reported yield of about 30%–40%.

Hydrogenation has been applied in mesophase preparation by reducing intermolecular interactions [96]. Pitch was first deashed, distilled, and then hydrogenated at 380–500 °C by using tetrahydroquinoline. The following heating eliminated the volatile fractions and formed a premesophase. Although the produced premesophase pitch was optically isotropic at the spinning temperature, it quickly oriented in the subsequent heating steps. For this method, coal pitches are preferred over petroleum pitches. Dormant mesophase was prepared by hydrogenating anisotropic pitch containing several percent of mesophase and heating the hydrogenated pitch below 380 °C [97]. It had large mesophase molecules, but a low softening point. The low softening point was due to the hydrogenation. Dormant mesophase oriented itself in heating after spinning. However, hydrogenation increased the production cost, and diminished the advantage of pitch as a low cost precursor.

Conoco Inc. (Ponca City, OK, U.S.A.) [98,99] has developed a solvated mesophase pitch. The solvated mesophase contains a small percentage (4–40 wt %) of solvent in the liquid crystalline structure. Therefore, it melts or fuses at a lower temperature providing the ease of spinning, while the spun fibers have much higher melting points when the solvent is removed. This high fiber melting point ensures little or no stabilization as spun. With aromatic solvents in the mesophase pitch, it has been reported that the 100% anisotropic pitch was very fluid at the 233 °C, while the residue at 650 °C when heated up at 5 °C/min showed no evidence of melting [98].

Yamada *et al*. [100] patented a process of preparing mesophase pitch from coal tar in the presence of a cracking catalyst. Coal tar pitch was first heated with an aromatic oil as the solvent, and silica-alumina or zeolite as a cracking catalyst at 350–500 °C for 10–60 min. After the removal of the insoluble materials, the pitch was heated at a temperature of 430–600 °C for less than 60 min. It was reported that the obtained pitch had good spinnability and the carbon fibers prepared from the pitch showed excellent physical properties and good mechanical properties.

Synthetic pitch prepared from the polymerization of naphthalene and its derivatives has attracted much attention recently. The synthetic pitch can be produced by polymerizing naphthalene, and/or its derivatives, with a Lewis acid as a catalyst [101]. In a patent by Seo, Oono and Murakami [102], naphthalene pitch was polymerized in the presence of AlCl_3_ at 150–300 °C. Water was added to remove the residual catalyst in the pitch to achieve high purity. A stable water-in-oil emulsion could be formed while stirring. The optimum viscosity while water was added was 10–150 centipoise. Mochida *et al*. [103,104] used HF/BF_3_ as a catalyst to polymerize naphthalene- and methyl-naphthalene-derived pitches. The naphthalene heated at 260–300 °C under pressure yielded pitches having 100% anisotropy and 215–285 °C softening points. They observed that the use of HF/BF_3_ greatly reduced the molecular weight distribution of the mesophase pitch. Spun fibers were stabilized at 270 °C in 15–30 min. The existence of methyl groups lowered the softening point and improved the spinnability and stabilization reactivity. Stabilization could be finished within 10 minutes at 270 °C. Mitsubishi Gas Chemical Co. has commercialized the production of naphthalene pitch with this HF/BF_3_ catalysis. Mesophase synthetic pitch can be produced following processes similar to those used for natural pitch. Compared with natural pitch, synthetic pitch has a lower softening point. Therefore, it can be extruded at a lower temperature to avoid the molecular decomposition and the formation of solid particles in spinning. Stabilization must be performed at a lower temperature, but can be at a significantly faster rate at a given temperature compared with anisotropic-isotropic mixtures of natural pitch. These could significantly reduce the processing cost. Compared with natural mesophase pitch, synthetic naphthalene pitch shows similar molecular weight distributions for anisotropic and isotropic fractions [105,106]. Thus a stable spinning can be achieved more easily. Isotropic pitch containing smaller mesophase spheres was believed to give smaller domains of random orientation in the fiber transverse direction, which could result in higher fiber mechanical properties [105,106]. Kamatsu *et al*. [105] prepared two naphthalene pitches containing mesophase spheres with different diameters at 360 °C and 375 °C, respectively. The resulting carbon fibers from the pitch with finer spheres showed smaller microdomains and thinner fibrils, and exhibited a higher compressive strength of 710 MPa [105]. The authors did not report the fiber tensile properties. In another article by the same research group [106], naphthalene pitch with about 50 vol % anisotropic content produced from EP-184 from Mitsubishi Gas Chemical Co. was processed into carbon fibers. The fibers showed a 516 GPa Young’s modulus and a 2.5 GPa tensile strength.

As mentioned earlier, mesophase pitch can be melt spun into precursor fibers. Compared with isotropic pitch spinning, this process needs a higher spinning temperature due to the higher anisotropic content and higher molecular weights. The spinneret needs to be vented to avoid gas bubble formation during spinning. The viscosities of both isotropic and mesophase pitches heavily depend on the temperature and the shear rates [1]. Compared with synthetic melt spinnable polymers, this high dependency on temperature creates a significantly higher tensile stress in spinning filaments [1]. The extruded filaments solidify in a very short distance below spinneret, which creates a large velocity gradient in the axial direction. Thus, tension can be formed on the filaments. Some variations, such as temperature gradient across the spinneret face and quenching air speed and temperature, can make the spinning more difficult. An accurate control on the spinning conditions is required to melt spin pitch precursors, which increases the processing cost. MP carbon fibers usually have a larger diameter of about 10–15 μm compared with the 5–7 μm diameter for PAN carbon fibers. This is because the MP precursor fibers with relatively large diameters are preferred to avoid fiber breakage during spinning. The other reason is pitch precursors have a higher carbon yield, thus the size reduction in carbonization/graphitization is smaller. A MP carbon fiber processing speed of up to 1,000 m/min can be achieved.

In another investigation, the spinning of mesophase pitch was modified by spinning the pitch upward [5,107]. The spinneret was embedded in a liquid (LiCl or KCl) (175–450 °C) heavier than the pitch precursor. The top layer of the liquid was at a higher temperature (500–650 °C) thus having a lower density. Due to the density difference, pitch fibers moved upward in the liquid and were dehydrogenated in the higher-temperature liquid layer. Inert atmosphere was on the top of the liquid for carbonization. This process could remove the oxidation step, but no data on extrusion speed and fiber properties has been reported. A high speed melt blown process has also been investigated to produce carbon fibers [108,109]. Airflow normal to the direction of the filaments is used to attenuate the fibers. The authors believed that this process could produce low-cost carbon fibers due to the relatively low-cost mesophase pitch, the high carbon yield of the pitch, and the high speed fiber spinning method [108,109].

In order to reduce fiber sticking or fusion and prevent fiber breakage or fluff, silicone oils or fine solid particles including graphite, carbon black, calcium carbonate, oxides, carbides *etc*., can be applied on the surface of the precursor fibers before stabilization to improve separability [5,110].

### 4.2. Thermal Treatment

Similar to PAN precursor fibers, the pitch fibers are infusibilized or oxidized in air at elevated temperatures before being exposed to the final high temperature carbonization treatment. The oxidization temperature should be below the fiber softening point to keep the orientated structure. Depending on the composition, mesophase pitch precursor is stabilized in air at 250–350 °C for a time ranging from 30 min to several hours [1,6]. There is no consent on the function of fiber stretching in this step. The oxidized pitch molecules contain ketone, carbonyl, and carboxyl groups that lead to the formation of a stronger hydrogen bonding between adjacent molecules. The introduction of oxygen containing groups and the formation of hydrogen bonding between molecules facilitate the three-dimensional crosslinking, but hinder the growth of crystallites [5]. Iodine has been used to reduce the stabilization time and increase the carbon yield for carbon fibers from natural pitch [111,112]. In a patent by Sasaki and Sawaki [111], the pitch fiber was soaked in a methanol solution of iodine till at least 0.05 wt % of iodine was imbibed. The fiber was then heated under an oxidizing atmosphere for infusibilization. The infusibilization time was affected by the amount of imbibed iodine but generally could be finished within approximately 10 min.

Stabilized fibers are then carbonized and graphitized. The greatest weight loss takes place in the early stages of carbonization. In order to avoid the defects created by the excessive release of volatiles, the fibers are preferred to be pre-carbonized for a brief period of 0.5–5 min at 700–900 °C. Carbon fibers can be produced by carbonizing the stabilized fibers to 1,500–1,800 °C. Bright and Singer [113] reported that due to the degradation of the structure, the modulus decreased at temperatures up to about 1,000 °C, but increased significantly upon further increase in temperature. Carbon fibers can be graphitized at temperatures close to 3,000 °C for improved Young’s modulus. Barr *et al*. [85] has shown that increasing heat treatment temperature could increase the preferred alignment of crystalline lamellae.

Although graphite layers are aligned along the fiber axis, the transverse structures of a carbon fiber can be different. Velocity gradients orient the layers radially, circumferentially or randomly. It has been reported that a radial crack can form in mesophase carbon fibers with layer planes distributed radially [4]. The alignments in the precursor fiber are retained in the resultant carbon fiber. Therefore, carbon fiber strength could be improved by adjusting the microstructure in the precursor fiber. Research has shown that the flaw sensitivity of MP carbon fibers is reduced by varying the microstructure of the pitch precursor fibers [114]. The microstructure can be modified by changing flow profiles during melting spinning [1]. A radial cross-section is usually formed through the laminar flow of the pitch melt [5]. Petoca Oil Company has used a technique of agitation in the spinneret to impart a randomized distribution for the folded graphene layer planes in the transverse direction [22,115,116]. The agitation created a turbulent flow and the produced carbon fibers showed increased tensile strength. The turbulent flow can also be obtained by different die designs since the flow behavior heavily depends on the shape of the spinneret [117,118]. Spinnerets containing sections with different diameters have shown to be able to change the fiber microstructure [119]. Since melt flow is dependent on melt viscosity, the change in microstructure can also be obtained simply by changing the spinning temperature. Otani and Oya [119] have shown that when the spinning temperature was raised to above 349 °C, the radial structure changed to either a random-type or a radial-type surrounded by an onion-skin type structure.

## 5. Cellulosic Carbon Fibers

The application of cellulosic fibers including cotton, flax, sisal, and linen, and regenerated continuous fibers as carbon fiber precursors has been studied. Among them, rayon has been used commercially and investigated most extensively. Rayon is produced from the least expensive cellulose, wood pulp by solution spinning. Insoluble cellulose is treated with NaOH and CS_2_ to form a soluble spinning dope. The xanthated cellulose is spun into fibers and recovered from the coagulation bath. Although rayon fabrics are still being used to convert into carbon fiber cloth, the production of carbon fibers from rayon has limited commercial values due to its low carbon yield (20%–30%), high processing cost, and limited physical properties [4,120]. The research has mostly been focused on modifying the degradation mechanism to increase the carbon yield.

Very similar to the case of PAN and MP carbon fibers, the conversion of rayon to carbon fiber includes thermal decomposition/oxidization, carbonization, and an optional graphitization. Rayon has a chemical formula of (C_6_H_10_O_5_)_n_ and the theoretical carbon yield is 44.4%. However, due to the releasing of CO, CO_2_ and other carbon containing gases in the process, the actual yield is only 10%–30%.

**Figure 6:**
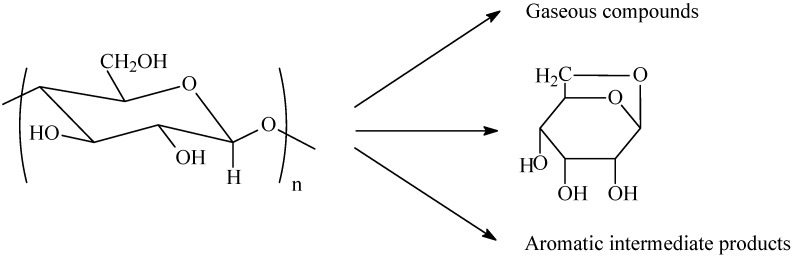
Thermal decomposition of cellulose.

Significant weight loss and structural changes can be observed in the thermal decomposition step. There are a great amount of reactions involved in cellulose decomposition/pyrolysis. The pyrolysis is affected by the crystalline fraction, molecular weight and conformation, heating medium, heating rate, and other factors [20]. The products can be classified into gaseous compounds, resins mainly formed from levoglucosan, and solid aromatic residues as shown in Figure 6 [20]. Finally, the aromatic residues are carbonized into carbon fiber.

In this thermal decomposition step, cellulosic units are first dried (<120 °C) and dehydrated (>120 °C) in air. Both inter- and intramolecular dehydration with the participation of hydroxyl groups contributes to the decrease in the hydroxyl peak in the infrared spectrum [67]. The intermolecular dehydration is preferred because a polymer network can be formed with the formation of intermolecular ether bond, which enhances the thermal stability. However, with the formation of C=O and C=C, the majority of dehydration reaction has been proposed to be intramolecular [6]. Chain scission at different sites along molecules is followed and the molecular weight is reduced significantly. One of the major reactions is the destruction of the 1,4 glycosidic bond and levoglucosan can be formed at one end of the cellulose molecules. The formation of levoglucosan reduces carbon yield and thus is an undesirable reaction [20]. Thermal dehydration and the formation of levoglucosan are competitive reactions. Dehydration starts at about 120 °C while the formation of levoglucosan takes place at above 250 °C [20]. Therefore, the control of preheating temperature can optimize the carbon yield of the final carbon fibers. Based on the thermogravimetric analysis results, most of the weight is lost in the range of 300–350 °C and the decomposition process depends on factors such as heating rate, fiber structure, and heating medium [20]. In the decomposition step, the degree of polymerization decreases but the content of carbon increases. After the elimination of CO and CO_2_, the four-carbon residues from each cellulose unit form carbon chains resulting in the formation of an aromatic structure upon high temperature heat treatment [6,121]. Aromatization and other structural changes mainly contribute to the exotherm at temperatures above 350 °C [20].

For rayon fibers without pretreatment, the thermal decomposition takes hours to finish with the temperature increasing slowly from room temperature to around 400 °C. Higher treatment temperature improves the diffusion of reactive gases into cellulose fibers and shortens the pyrolysis step. However, the unfavorable levoglucosan is produced in a larger amount resulting in a lower carbon yield. The application of flame retardants, such as Lewis acids, bases, strong acids and halides, can promote the dehydration and reduce the amount of levoglucosan produced and thus reduces the treatment time frame from hours to minutes [122]. Flame retardants can be applied through impregnating the fibers in the aqueous dispersions of flame retardants. With the application of flame retardants, the dehydration starts at a lower temperature [123]. The maximum rate of weight loss is also observed in a lower temperature range [123]. Therefore, thermal decomposition takes place with a less amount of levoglucosan produced but at a higher pyrolitic rate. Preoxidization of cellulose can also increase carbon yield [20]. At the early stages of oxidation, aldehyde or ketone groups are formed, which favors the intermolecular crosslinking reactions. Pyrolysis in the presence of an active medium (e.g., O_2_) has shown to be able to oxidize the C6 methylol groups [6]. Therefore, the amount of released levoglucosan is reduced and the aromatic intermediate products for carbon fibers are increased accordingly. The rate of pyrolysis is higher in the low ordered (amorphous) region, partially due to the easy oxygen diffusion. It has also been reported that the use of HCl started the decomposition at a lower temperature and increased the carbon yield [124]. The carbon yield was about 35% when the thermal treatment temperature was around 1,000 °C. The carbon content of the stabilized precursors is usually 60%–70%.

In the carbonization step, the carbon content is further increased. The temperature range for this step is roughly from 400 °C to 1,500 °C and it could take tens of hours through increasing the temperature slowly. As temperature increases, the carbon content increases but the chemical complexity also increases due to the vast amount of different bonds formed between carbon atoms [20]. The process is conducted in an inert atmosphere, like nitrogen. In another research, the application of 1–2 vol % of oxygen was described [125]. The pressure was kept at less than 50 mm Hg and fibers were treated up to 1,200 °C. It was observed that oxygen burned off the unorganized carbon, thus improved the mechanical properties of the carbon fibers.

The carbon fibers can be graphitized at even higher temperatures in the range of 1,500–2,500 °C to graphite fibers. Carbon content is increased to above 99% and the fiber density is usually increased due to the growth of crystallites. The duration of graphitization is in the order of seconds and can be less than one second depending on the treatment temperature. The Young’s modulus increases with the treatment temperature if the graphitization is conducted under tension.

Rayon fibers are stretched during carbonization and graphitization to achieve the orientation of carbon layers for improved properties. In this case, stretching in the stabilization step is not as effective as stretching at the early stages of carbonization. It is partially due to the low strength of the decomposed cellulose fibers. Stretching changes the reaction kinetics and the loss of weight can be increased significantly if cellulose is stabilized under tension. The production of rayon carbon fiber is expensive because of this costly high-temperature stretching treatment. With an effective stretching of around 100% in an inert atmosphere, the graphitic layer planes are aligned in the fiber axial direction and the elastic modulus of the resultant carbon fibers can be more than 500 GPa with a medium tensile strength of around 2.5 GPa [122].

## 6. Carbon Fibers Made from Other Precursors

### 6.1. Lignin—The Efforts on Cost Reduction

Researchers continue to look for carbon fiber precursors with low cost, high carbon content and biorenewablity. The major contributors to the high cost of carbon fibers are the precursors and the capital equipment for the conversion of the precursor. Biorenewable lignin has been investigated as a potential precursor material for carbon fiber as it is the most abundant phenolic compounds in nature and is produced as the byproduct of the pulping process and cellulosic ethanol fuel production. The dominant chemical pulping process nowadays is the kraft process using a solution of sodium hydroxide and sodium sulfide. As one of the major applications, the lignin solution is concentrated and burned to supply energy and to facilitate the recovery of the pulping salts. In nature, lignin is a polyaromatic polymer consisting of phenylpropane units. Lignin obtained from the pulping process has usually undergone a hydrolytic degradation depending on the pulp sources and pulping processes.

Otani *et al*. [126] patented a process to manufacture carbon fibers from lignin in as early as 1969. Sudo *et al*. [127,128] also produced carbon fibers from lignin with fairly good mechanical properties by breaking intermolecular interactions through modifying hydroxyl and hydroxy methyl groups.

Sano *et al*. [129,130] reported a process of producing carbon fibers from an organosolv lignin. The lignin is obtained from birch wood by an aqueous acetic acid pulping process. Organosolv lignin is much purer than the commercial kraft lignin. Once removing the volatile components, the lignin shows nice melt spinnability. The carbon fibers with a 14 ± 1 μm diameter showed tensile strength of about 355 MPa and modulus of about 39.1 GPa.

Kadla *et al*. [131] produced carbon fibers from a hardwood kraft lignin, an organosolv lignin, and blends of lignin and poly(ethylene oxide) (PEO). The kraft lignin was first purified or desalted by repeated washing using distilled water while keeping the pH below 5 with HCl till the salt was less than 1,000 ppm. Precursors were then extruded at temperatures between 130 °C and 240 °C depending on the composition of the extrudates. It was shown that PEO was miscible with the lignin and facilitated the fiber spinning. The precursor fibers were then stabilized at temperatures up to 250 °C in air and carbonized in nitrogen at the final temperature of 1,000 °C. The carbon yield was between 40%–46%. The tensile strength and the modulus of the resultant carbon fibers (31–63 μm diameter) were in the range of 400–550 MPa and 30–60 GPa, respectively. No stretching was applied in the heating process.

Kubo and Kadla [132] produced carbon fibers from a blend of hardwood lignin and poly(ethylene terephthalate) (PET) using a similar process as described above. The carbon fiber yield decreased with the increased amount of PET. Upon blending with PET, the mechanical properties of the carbon fibers increased slightly. For the precursor with a lignin/PET ratio of 75/25, the resultant carbon fibers with a diameter of about 34 ± 5μm showed modulus of 94 GPa and tensile strength of about 703 MPa.

Kadla *et al*. [133] also studied the stabilization of lignin precursor fibers in air at various constant heating rates by building a continuous heating transformation diagram. A heating rate of 0.06 °C/min was required to ensure Tg was above the temperature of the heating medium. Elemental analysis revealed that carbon and hydrogen content decreased in the whole stabilization step while the oxygen content increased at temperatures of up to 200–250 °C and then decreased at higher temperatures. At the earlier stages of stabilization, free radicals were produced by the cleavage of ether bonds including arylglycerol-β-O-4 aryl ether bonds. The chemical reaction of free radicals in the presence of oxygen introduced ketones, phenols, and possibly carboxyl groups [134,135,136]. With the further increase in temperature, the loss of mass was caused by the release of CO_2_ and water, and the formation of anhydrides and possibly esters [133]. The authors also reported that aromatic carbon–carbon bonds were formed at high temperatures.

ORNL researchers have conducted a lot of research on the application of alkaline lignin as a carbon fiber precursor. To date, the purification process has been very costly (roughly $1/lb). MeadWestvaco (Charleston, SC) is developing a low cost organic purification process to control impurity levels in kraft lignin [46]. ORNL researchers have shown that the purified hardwood lignin is readily melt-spinnable. However, its melting point is about 130 °C thus low temperature stabilization is required, which reduces the stabilization rate significantly [46]. Softwood lignin has shown to have a better potential as its carbon yield is higher than that of hardwood lignin. However, softwood lignin has a more cross-linked structure, thus it is not melt spinnable without modification [46]. By blending with plasticizers, ORNL researchers have shown that softwood lignin can be melt spun into precursor fibers [46]. A precursor fiber with a diameter of 10 μm was extruded at ORNL by blending a hardwood lignin with a softwood lignin. It is indicated that the carbon yield has approached 50% and heat stretching during the thermal treatment could further improve the carbon fiber mechanical properties.

### 6.2. Other Precursor Materials

Many other polymers have also been investigated for their potential as carbon fiber precursors. In addition to cellulosic fibers discussed earlier, other natural fibers have been investigated including silk [137], chitosan [138] and eucalyptus tar pitch [139]. They have the potential of lowering production cost. However, most of them are used for general purpose carbon fibers which do not provide high mechanical properties.

Synthetic polymers have also been evaluated as alternative carbon fiber precursors. General Electric patented a continuous process to convert polyacetylene fibers to carbon fibers [140]. Precursor fibers were solution spun and coagulated in an acetone bath. The coagulated fiber tow was then treated for stabilization at 260 °C and 360 °C, respectively. The carbonization was conducted at 1,100 °C. The carbon fibers were further graphitized at a constant strain of 100% at 2,500 °C for about 2 min to achieve high mechanical properties. The carbon fibers showed a Young’s modulus in the range of 350–490 GPa.

A melt extrudable mixture of a polyacetylene copolymer was also disclosed [141]. The polyacetylene used in this work was made by the polymerization of meta-diethynylbenzene and para-diethynylbenzene together with dipropargyl ethers of dihydric phenols. The plasticizer was dichlorobenzene or pyridine. The melt spun fibers were drawn for increased orientation and decreased diameter. It was reported that the fibers with about 25 μm diameter could be stabilized at 310 °C for 0.5 sec and then at 200 °C and 300 °C for 20 sec either in the presence or absence of oxygen. The thermally stabilized fibers were converted to carbon fibers at 1,000 °C and the resultant carbon fibers showed a tensile strength of about 2.3 GPa and a modulus of about 386 GPa.

Pennings *et al*. [142] have investigated the potential preparation of carbon fibers from selectively polymerized poly(vinylacetylene) by the polymerization of vinyl groups in monovinylacetylene. The polymer was melt spinnable. The pendent acetyl group was responsible for the cyclization reactions to form a conjugated ladder structure similar to the nitrile in PAN stabilization. The precursor fibers were exposed to a 300 nm UV light source for a few hours in a nitrogen atmosphere, and then heated to 225 °C in nitrogen. Oxidation was conducted at 225 °C in air. The authors did not produce carbon fibers from this precursor.

A process of producing carbon fibers from polyethylene (PE) was disclosed in U.S. patent 4070446 [143]. Melt spun PE fibers were immersed in chlorosulfonic acid (or sulfuric acid, fuming sulfuric acid or a mixture) at 80 °C for about 90 min for sulfonation. The washed and dried fibers were then carbonized in argon by raising the temperature to 1,200 °C, while under a tension of 16 mg/denier. The carbonization yield was as high as 75% and, with the application of tension, the modulus and tensile strength were 139 GPa and 2.5 GPa, respectively.

Ashitaka *et al*. [144,145] developed carbon fibers from syndiotactic 1,2 poly(butadiene) (s-PB). The syndiotacticity induces the formation of thermally stable spiral ladder polymers. The s-PB fibers were prepared by melt spinning at 205 °C. The precursor fibers were stabilized by immersing into a solution of AlBr_3_ in benzene at 42 °C for 78 min under tension. The washed and dried fibers were then immersed into molten sulfur at 275 °C for 14 min for dehydrogenation. The adhering sulfur was purged with nitrogen at 290 °C for 7 min. Carbon fibers obtained by heating the precursors to 1,400 °C in an inert atmosphere showed a tensile strength of about 1.6 GPa and a modulus of about 139 GPa with a carbon yield of 82%. Carbon fibers obtained through heat treatments of up to 3,000 °C showed a tensile strength of about 2.0 GPa and a modulus of about 393 GPa with a carbon yield of 70%.

A similar process for making polybutadiene carbon fibers was described by Nagasaka *et al*. [146]. The melt-spinning polybutadiene fibers were cyclized and cross-linked in a solution or suspension of a Lewis acid in an inert organic liquid and then treated in a sulphur melt or a sulphur-containing solution at 170–300 °C for aromatization. The fibers were carbonized at 750–1,500 °C in nitrogen and graphitized at 1,500–3,000 °C in argon. The carbon fibers carbonized without tension had a high carbonization yield of about 89%, but relatively low mechanical properties of about 58–71 GPa modulus and 0.95–1.2 GPa tensile strength.

Edie *et al*. [147] obtained carbon fibers from poly (p-phenylene benzobisoxazole) (PBBO). PBBO can be carbonized through a regular process into carbon fibers without stabilization. However, the resulting carbon fiber shows low mechanical properties with a tensile strength of up to 1 GPa and a modulus of up to 245 GPa. They concluded that the flaws in the precursor fibers remained in the carbon fibers resulting in the low carbon fiber mechanical properties. The authors believed that the properties could be improved by modifying the PBBO precursor fiber spinning process.

Other linear and cyclic polymers investigated include phenolic polymers [148,149], polyacenephthalene [150], polyamide [151], polyphenylene [152], poly (p-phenylene benzobisthiazole) (PBBT) [153], polybenzoxazole [154], polybenzimidazole [155], polyvinyl alcohol [156], polyvinylidene chloride [157], polystyrene [158], and so on. Linear precursors require heat stretching to obtain high performance carbon fibers and their carbon yields are usually very low [6]. The polymers with a high aromatic content can generally offer a high carbon yield and in some cases, easy stabilization. But, these polymers either have high costs or do not produce high performance carbon fibers. Research needs to be conducted to reduce the processing cost while improving the mechanical properties of the resultant carbon fibers.

## 7. Future Work

The future efforts on carbon fiber research will be focused on cost reduction and property improvement. The mechanical property of carbon fiber heavily relies on its microstructure. The improvement on the tensile, flexural, and shear strength of pitch carbon fibers has been observed by randomizing the graphite distribution in the fiber transverse direction. The crystallite size and distribution can be changed by varying the die design, spinning temperature, spinning rate, PAN copolymer type and its stereoregularity, and other parameters. However, research in this area is not enough. The optimization of the crystallite size, shape, and distribution by changing processing parameters and their effect on the fiber properties need to be investigated. Future understanding of the relationship between the ratio of *sp^2^*/*sp^3^* bonding and the fiber property also has the potential to lead to improved fiber properties.

Preliminary investigations on reducing the processing cost by melt spinning of PAN have shown promising results. The melt assisted spinning process has produced carbon fibers with a tensile strength of about 3.6 GPa and a modulus of about 233 GPa. Further improvement on the fiber mechanical properties has been hindered by the existence of defects. The application of different plasticizers (besides water) and the modification of the process to minimize the defects created by the evaporation of plasticizers need to be investigated.

Compared with PAN, pitch is a low cost precursor. However, the processing cost is high, as the isotropic pitch needs to be transformed into a mesophase pitch for the production of high performance carbon fibers. Researchers have been active on reducing the cost for mesophase pitch preparation. The breakthrough in this field can significantly reduce the cost of pitch carbon fibers.

Many polymers have been evaluated as the low cost precursor materials. Lignin is a well known potential precursor for low cost and medium property carbon fibers. High-purity organosolv lignin can be spun into precursor fibers directly. Another promising low cost precursor material is PE. The carbon fiber made from PE has shown a high carbon yield of 75% and an appropriate strength of 2.5 GPa. However, not enough work has been done on the process optimization. The research on shortening the stabilization time through different methods (the addition of active chemicals in the spinning dope as an example) needs to be evaluated. The effect of heat stretching on the mechanical properties of the resultant carbon fibers also needs to be investigated.

## 8. Conclusions

Polyacrylonitrile (PAN) and mesophase pitch (MP) are the two most important carbon fiber precursors. A significant amount of work has been done on relating fiber structure to properties and translating the relationship into production for either reducing production cost or increasing fiber properties. However, challenges including cost reduction, tensile and compressive strength improvement, and alternative precursor development still remain.

Optimizing the carbon fiber microstructure can improve carbon fiber strength through decreasing its flaw sensitivity. The carbon fiber microstructure is dependent on the precursor morphology and processing conditions. Research in these two areas will aid in the development of carbon fibers with improved performance.

Many polymers, such as PE and lignin, have been evaluated as low cost carbon fiber precursor materials. It opens the door for low cost carbon fibers. However, more research needs to be conducted to optimize processing conditions to enhance the mechanical properties and carbon yield of the resultant carbon fibers.
